# Supplementary material for: An integrated multi-layer 3D-fabrication of PDA/RGD coated graphene loaded PCL nanoscaffold for peripheral nerve restoration
Source: Nat Commun. 2018 Jan 22;9:323. doi: 10.1038/s41467-017-02598-7 (PMC5778129; doi:10.1038/s41467-017-02598-7)
Supplement: Supplementary file 1 — Supplementary Information [file 41467_2017_2598_MOESM1_ESM.pdf]

Supporting Information

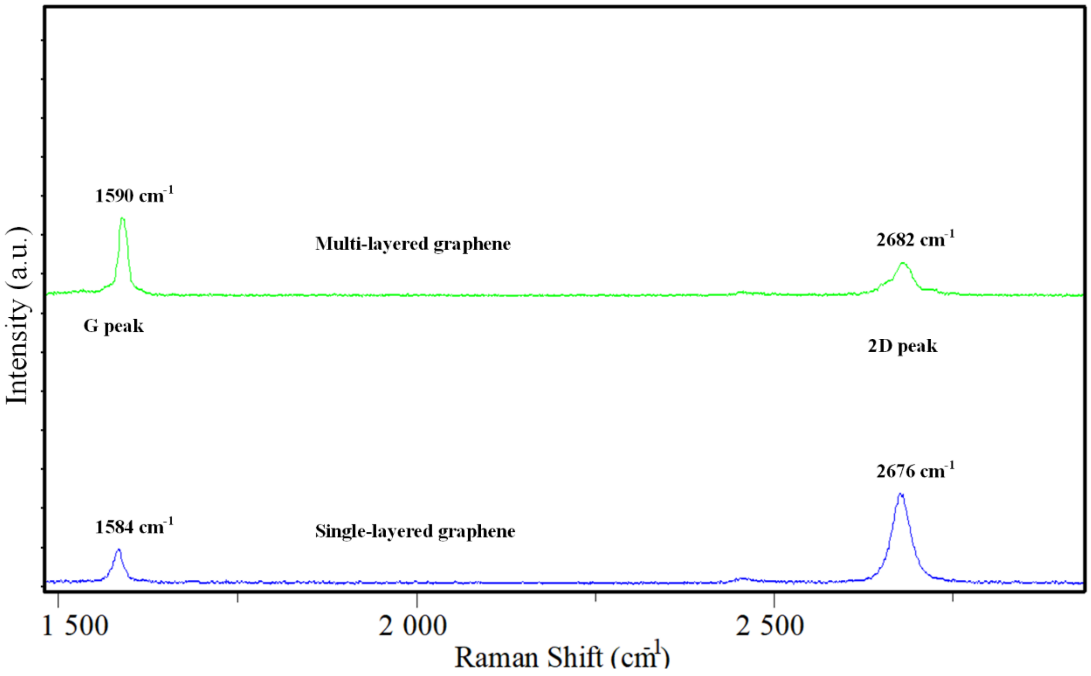

Supplementary Figure 1. Raman spectrum for single-layered and multi-layered graphene nanoparticles. The different shape, intensity and position of G peak and 2D peak were displayed from two materials.

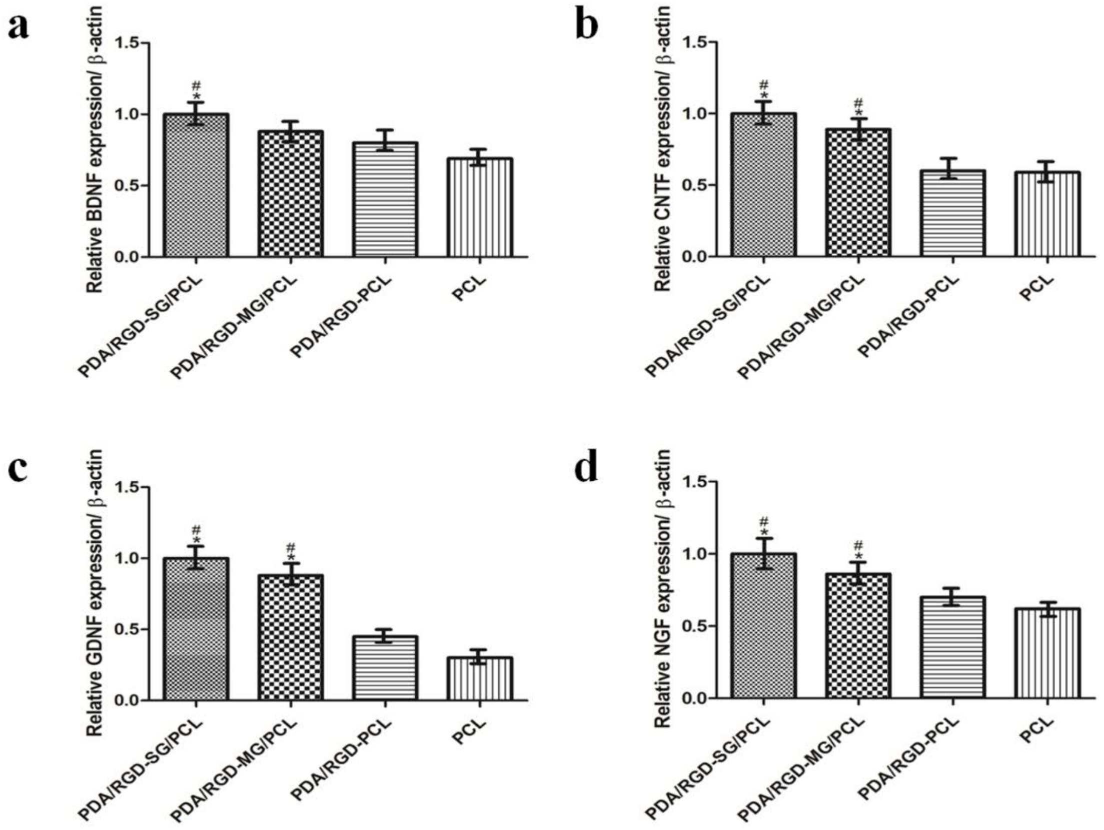

Supplementary Figure 2. **Quantitative PCR of BDNF, CNTF, GDNF, and NGF from SC on the different nanoscaffolds.** (a) Relative BDNF expression. (b) Relative CNTF expression. (c) Relative GDNF expression. (d) Relative NGF expression. All data are displayed as mean ± standard deviation. *$p<0.05$ compared with PDA/RGD-PCL; $^{\#}p<0.05$ compared with PCL (the statistical test is ANOVA).

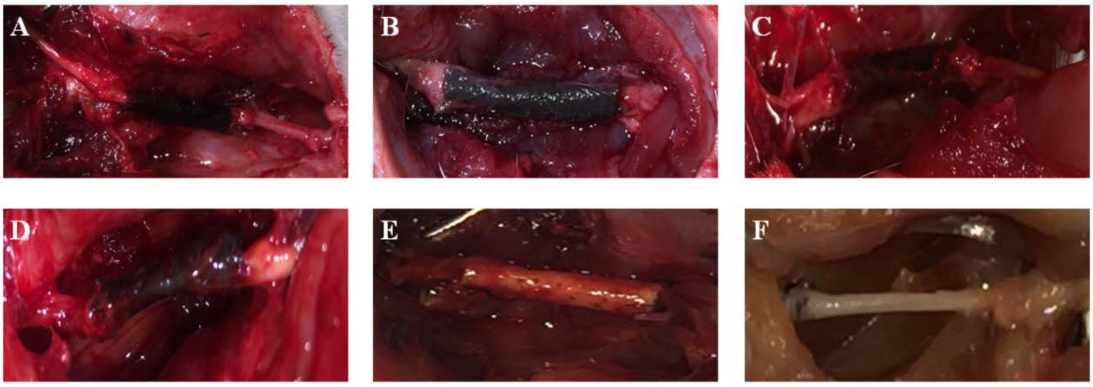

Supplementary Figure 3. **Regenerated nerves in different groups at 18 weeks after surgery.** SC loaded PDA/RGD-SG/PCL (A). SC loaded PDA/RGD-MG/PCL (B). PDA/RGD-SG/PCL (C). PDA/RGD-MG/PCL (D). PDA/RGD-PCL (E). Autograft (F).

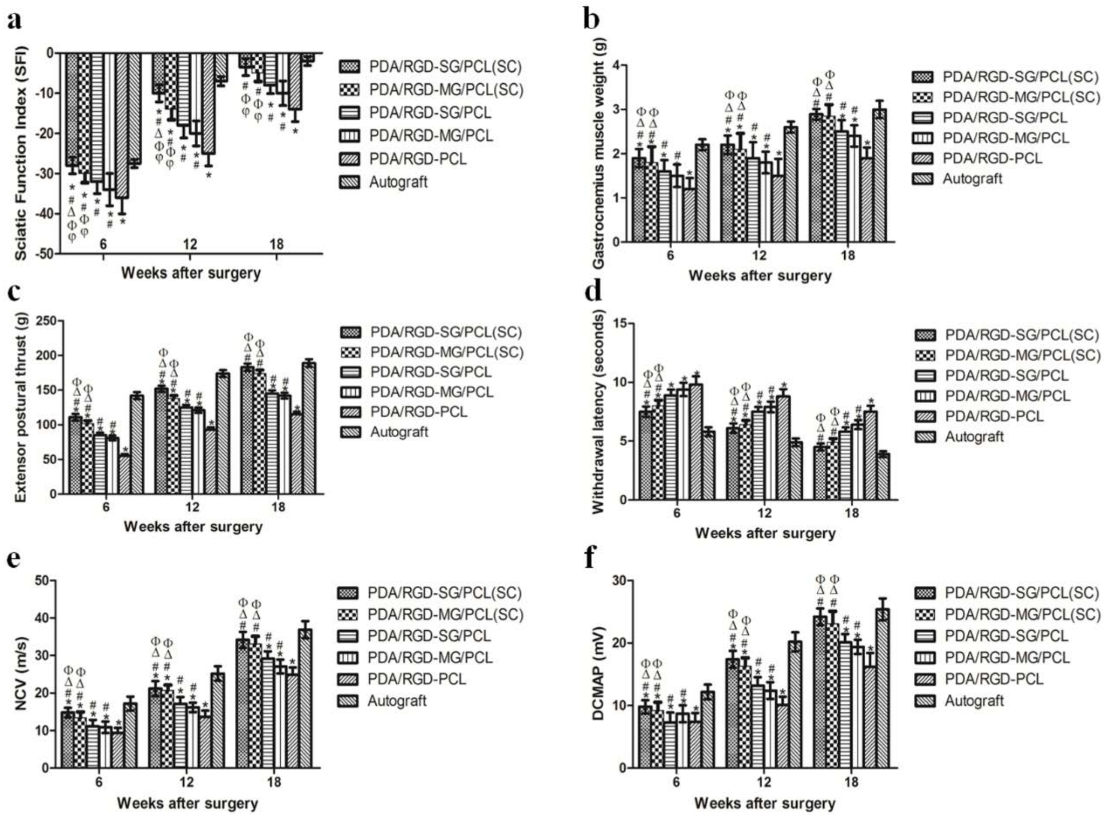

Supplementary Figure 4. Functional and electrophysiological assessment of nerve regeneration. (**a**) SFI. (**b**) Gastrocnemius muscle weight. (**c**) Extensor postural thrust. (**d**) Withdrawal latency. (**e**) NCV, (**f**) DCMAP. All data are displayed as mean ± standard deviation. *$p<0.05$ compared with autograft (n=5); $^{\#}p<0.05$ compared with PDA/RGD-PCL (n=5); $^{\Delta}p<0.05$ compared with PDA/RGD-MG/PCL (n=5); $^{\phi}p<0.05$ compared with PDA/RGD-SG/PCL (n=5) (the statistical test is ANOVA).

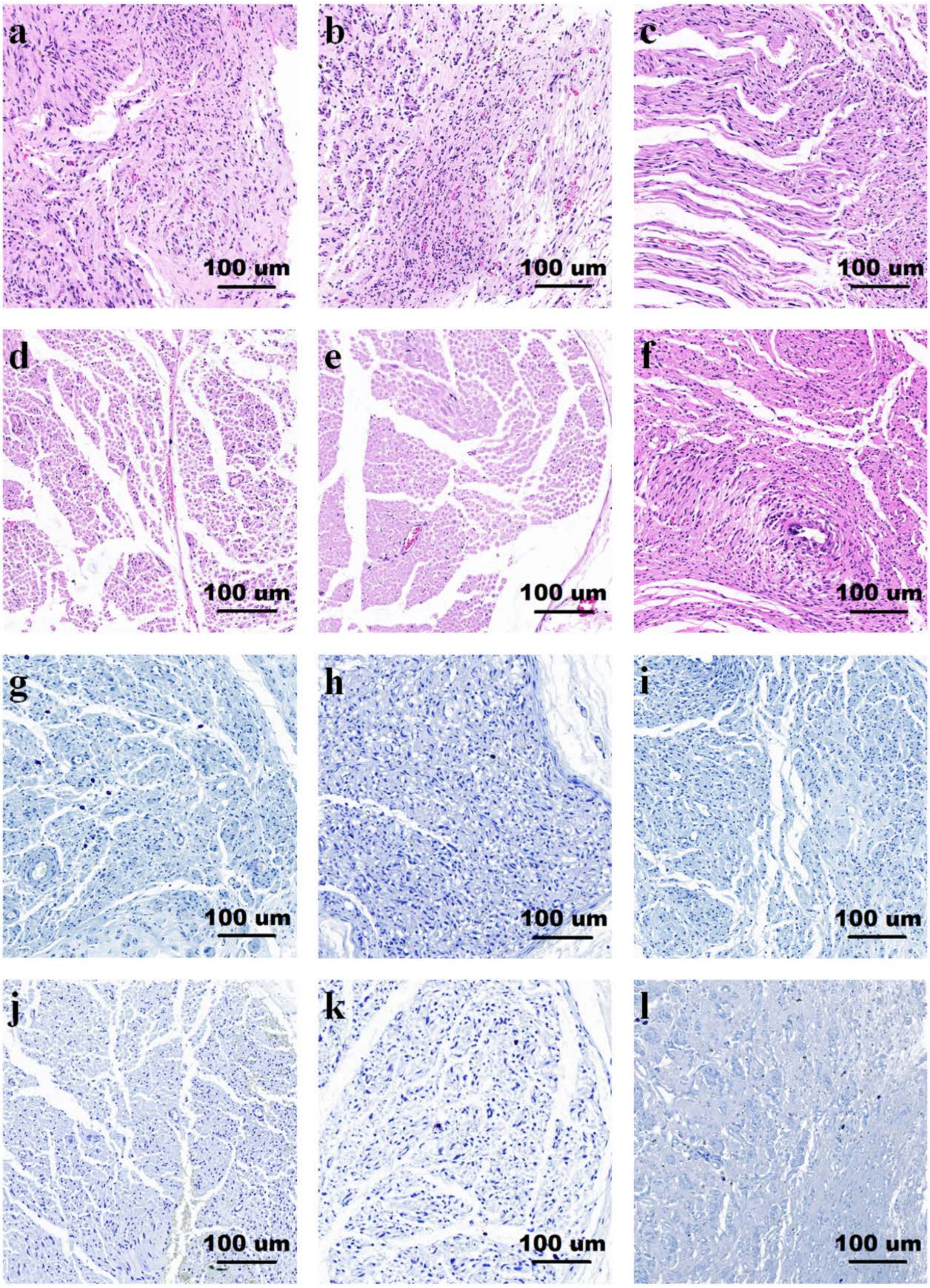

Supplementary Figure 5. (**a-f**) **HE staining and** (**g-l**) **TB staining for regenerated nerves from different groups at 6 weeks postoperatively.** (**a,g**) SC loaded PDA/RGD-SG/PCL. (**b,h**) SC loaded PDA/RGD-MG/PCL. (**c,i**) PDA/RGD-SG/PCL. (**d,j**) PDA/RGD-MG/PCL. (**e,k**) PDA/RGD-PCL. (**f,l**) Autograft. The scale bar is 100 μm.

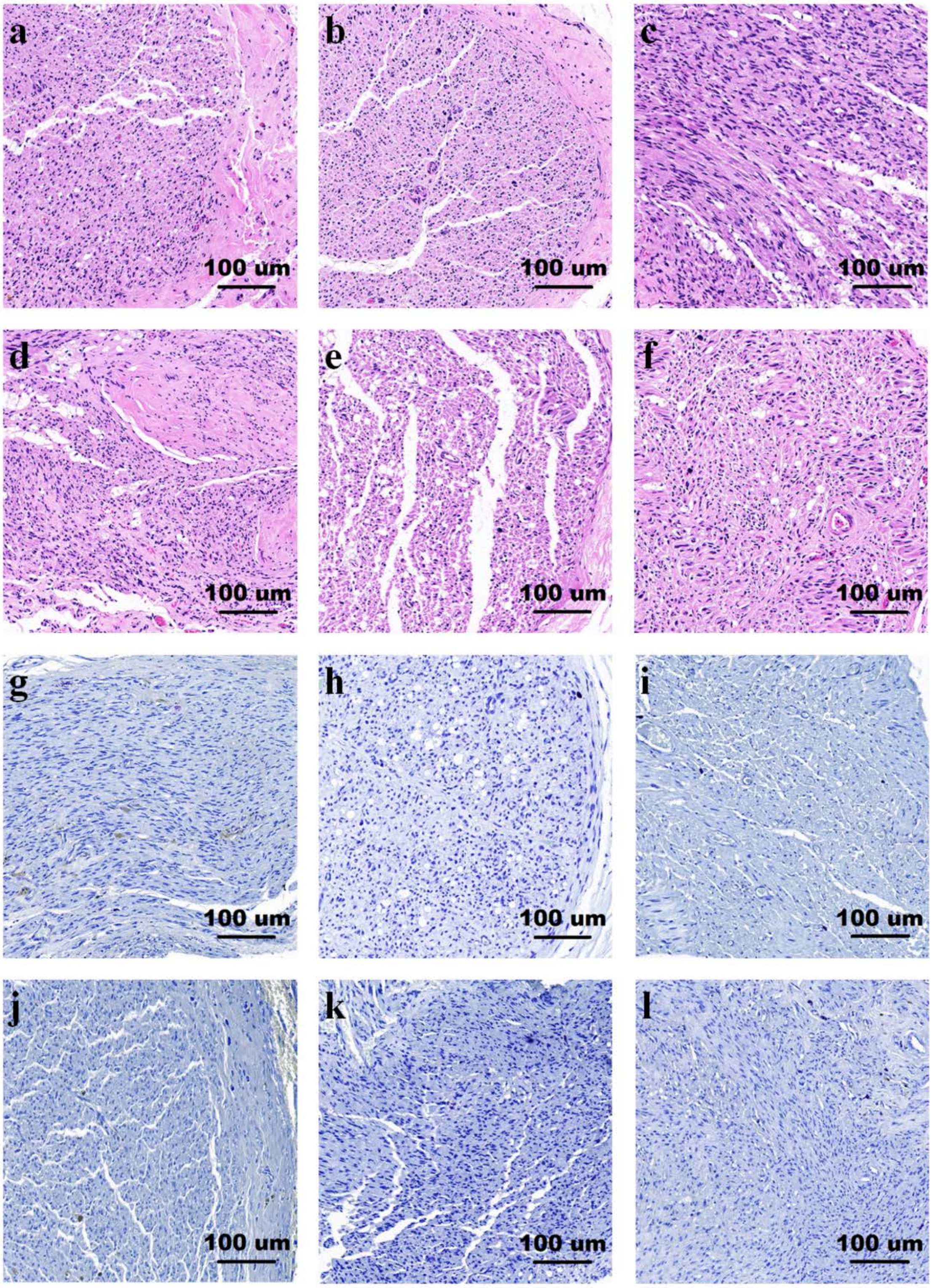

Supplementary Figure 6. (**a-f**) **HE staining and** (**g-l**) **TB staining for regenerated nerves from different groups at 12 weeks postoperatively.** (**a,g**) SC loaded PDA/RGD-SG/PCL. (**b,h**) SC loaded PDA/RGD-MG/PCL. (**c,i**) PDA/RGD-SG/PCL. (**d,j**) PDA/RGD-MG/PCL. (**e,k**) PDA/RGD-PCL. (**f,l**) Autograft. The scale bar is 100 μm.

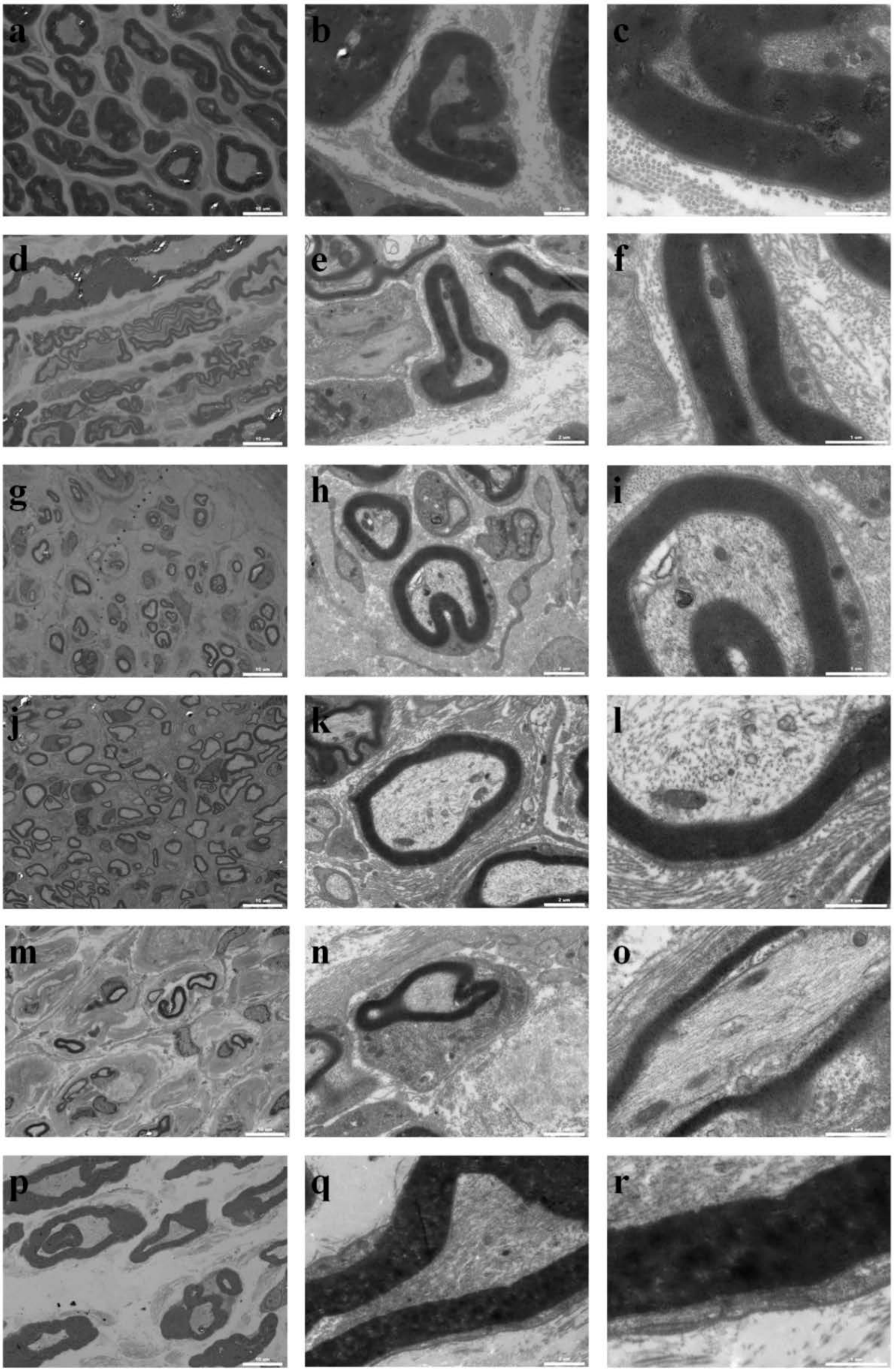

Supplementary Figure 7. TEM for regenerated myelinated axons from different groups at 6 weeks postoperatively. (**a-c**) SC loaded PDA/RGD-SG/PCL. (**d-f)** SC loaded PDA/RGD-MG/PCL. (**g-i**) PDA/RGD-SG/PCL. (**j-l**) PDA/RGD-MG/PCL. (**m-o**) PDA/RGD-PCL. (**p-r**) Autograft. The scale bar in a, d, g, j, m and p is 10 μm. The scale bar in b, e, h, k, n and q is 2 μm. The scale bar in c, f, i, l, o and r is 1 μm.

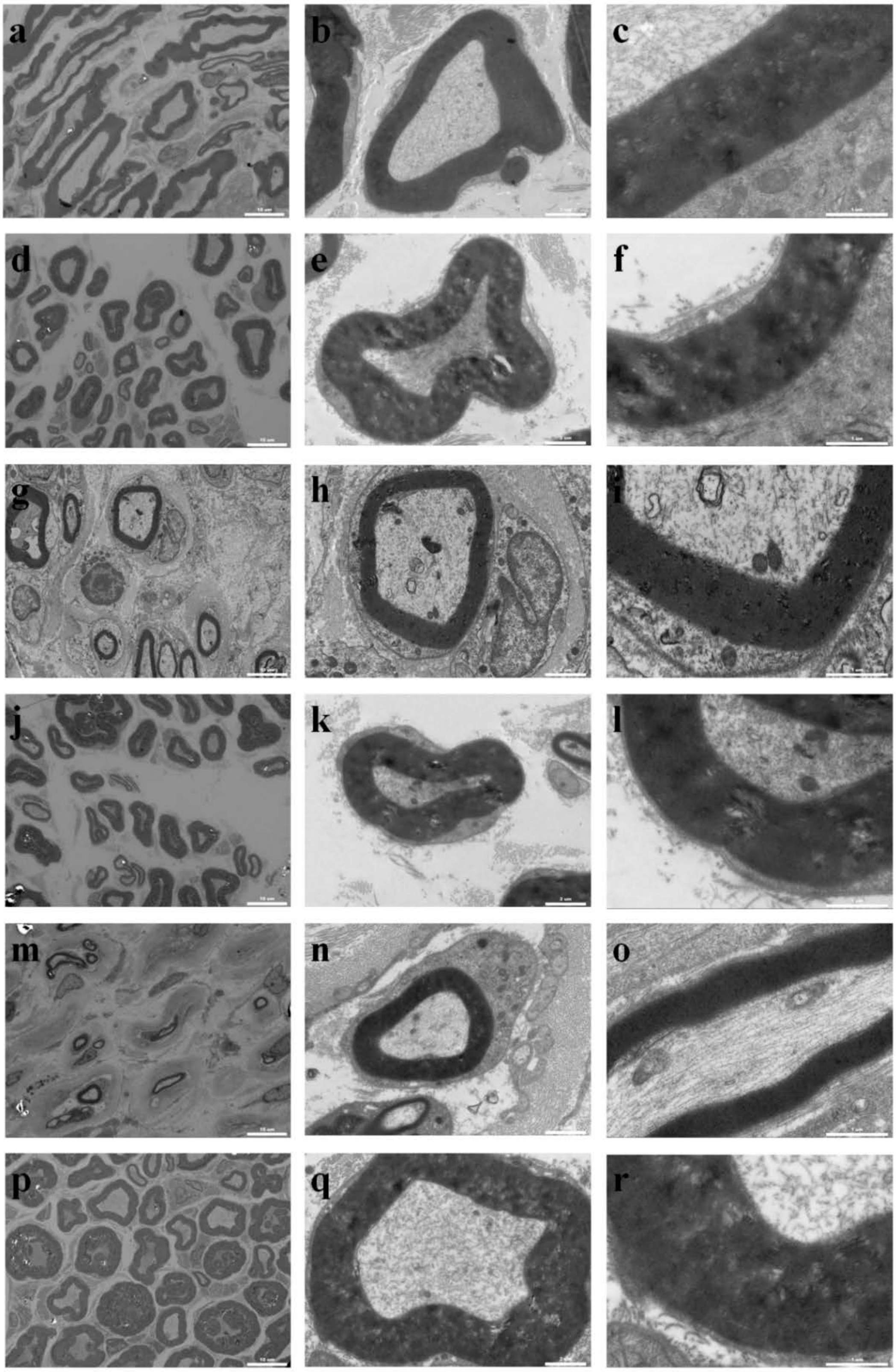

Supplementary Figure 8. TEM for regenerated myelinated axons from different groups at 12 weeks postoperatively. (**a-c**) SC loaded PDA/RGD-SG/PCL. (**d-f**) SC loaded PDA/RGD-MG/PCL. (**g-i**) PDA/RGD-SG/PCL. (**j-l**) PDA/RGD-MG/PCL. (**m-o**) PDA/RGD-PCL. (**p-r**) Autograft. The scale bar in a, d, g, j, m and p is 10 μm. The scale bar in b, e, h, k, n and q is 2 μm. The scale bar in c, f, i, l, o and r is 1 μm.

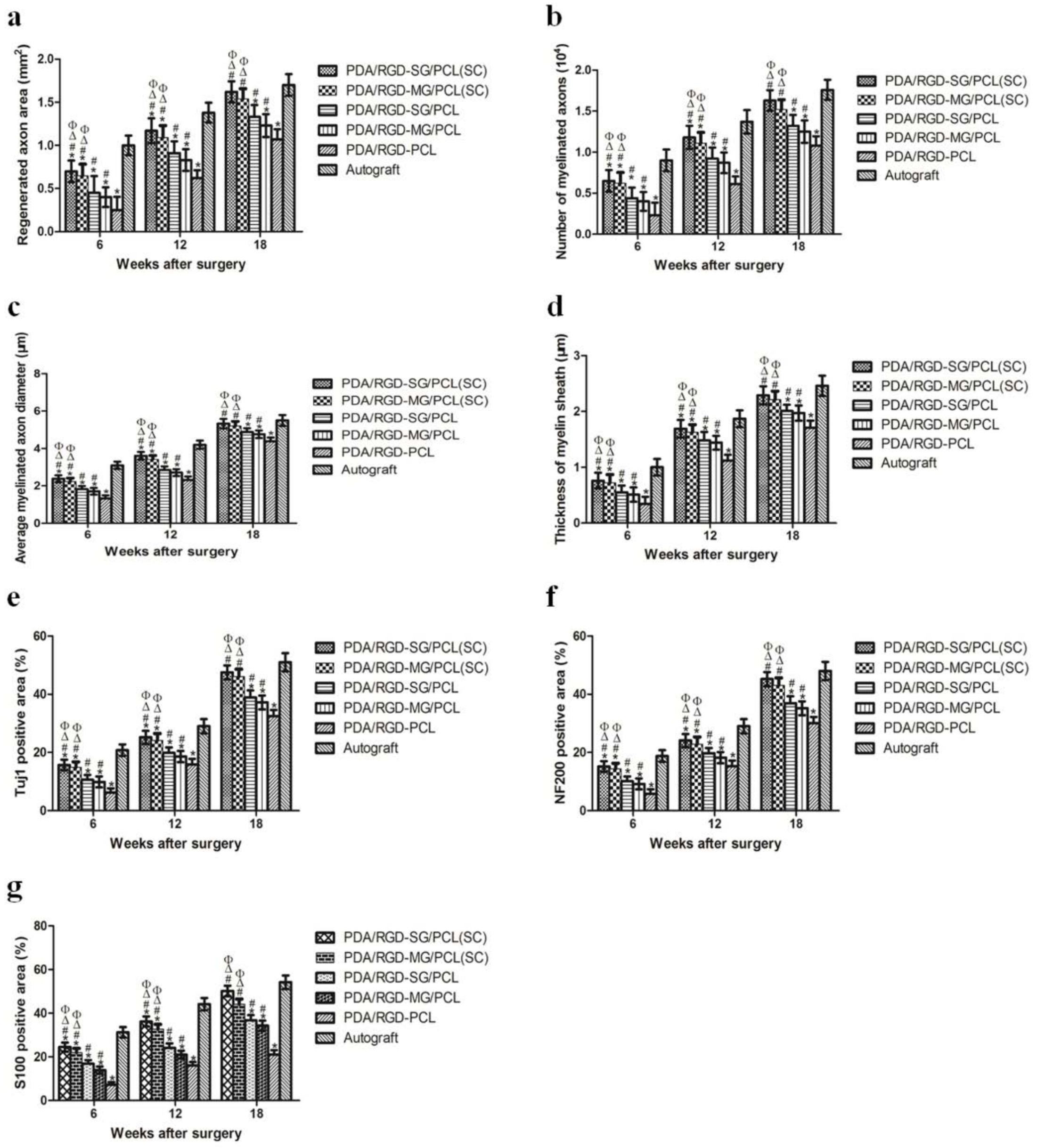

Supplementary Figure 9. Morphological evaluation of nerve regeneration. (**a**) Regenerated axon area. (**b**) Number of myelinated axons, (**c**) Average myelinated axon diameter. (**d**) Thickness of myelin sheath. (**e**) Tuj1 positive area (%). (**f**) NF200 positive area (%). (**g**) S100 positive area (%). All data are displayed as mean ± standard deviation. $^{*}p<0.05$ compared with autograft; $^{\#}p<0.05$ compared with PDA/RGD-PCL (n=5); $^{\Delta}p<0.05$ compared with PDA/RGD-MG/PCL (n=5); $^{\phi}p<0.05$ compared with PDA/RGD-SG/PCL (n=5) (the statistical test is ANOVA).

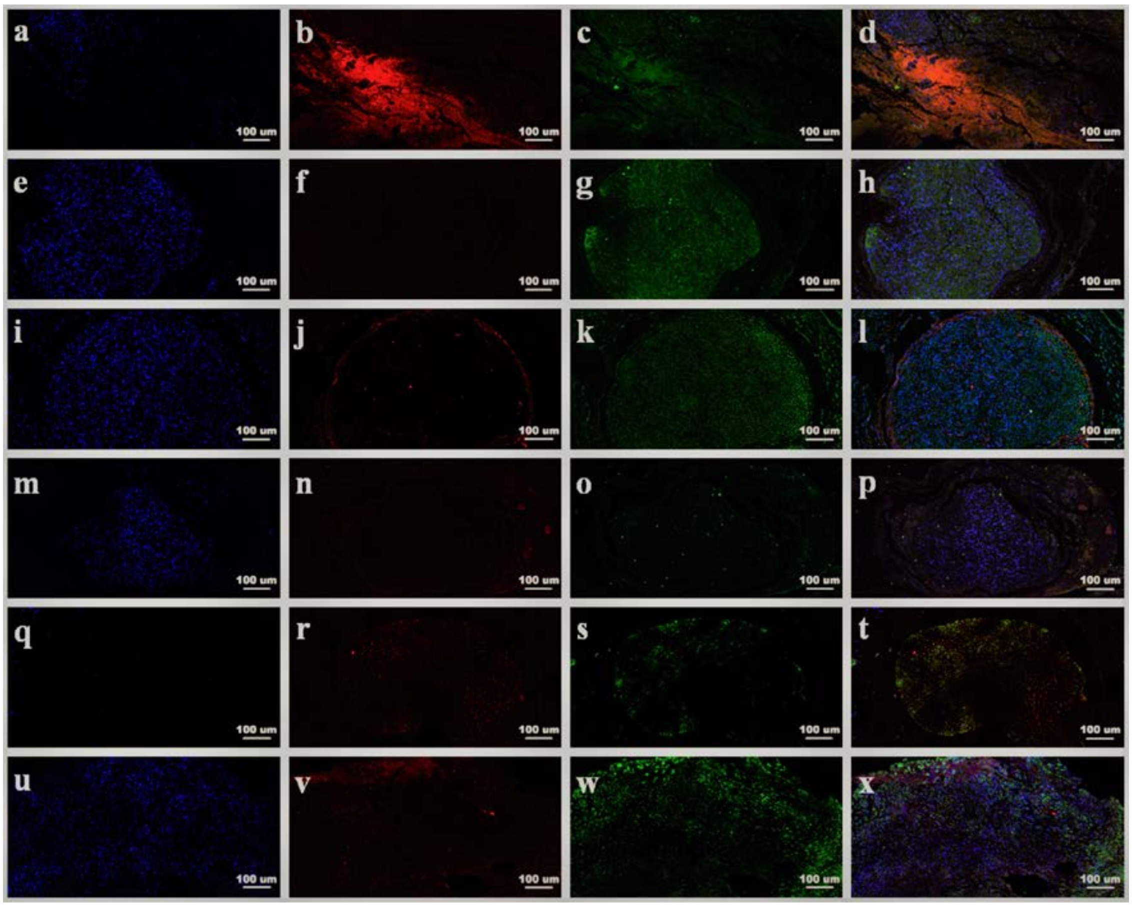

Supplementary Figure 10. Triple immunofluorescent staining of Tuj1 and NF200 of regenerated nerves at 6 weeks postoperatively. Tuj1 (green), NF200 (red) and nuclei (blue) were exhibited from different groups respectively. (**a-d**) SC loaded PDA/RGD-SG/PCL. (**e-h**) SC loaded PDA/RGD-MG/PCL. (**i-l**) PDA/RGD-SG/PCL. (**m-p**) PDA/RGD-MG/PCL. (**q-t**) PDA/RGD-PCL. (**u-x**) Autograft. The scale bar is 100 μm.

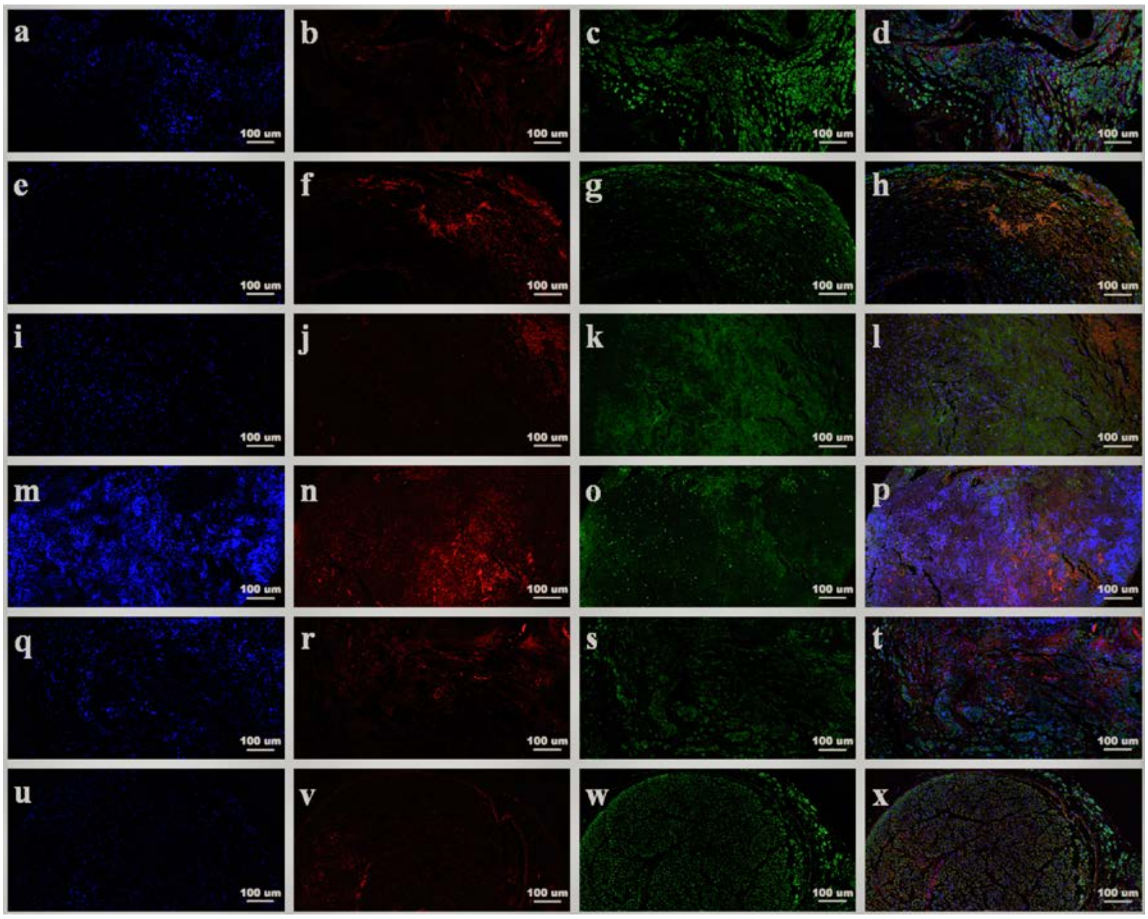

Supplementary Figure 11. Triple immunofluorescent staining of Tuj1 and NF200 of regenerated nerves at 12 weeks postoperatively. Tuj1 (green), NF200 (red) and nuclei (blue) were exhibited from different groups respectively. (**a-d**) SC loaded PDA/RGD-SG/PCL. (**e-h**) SC loaded PDA/RGD-MG/PCL. (**i-l**) PDA/RGD-SG/PCL. (**m-p**) PDA/RGD-MG/PCL. (**q-t**) PDA/RGD-PCL. (**u-x**) Autograft. The scale bar is 100 μm.

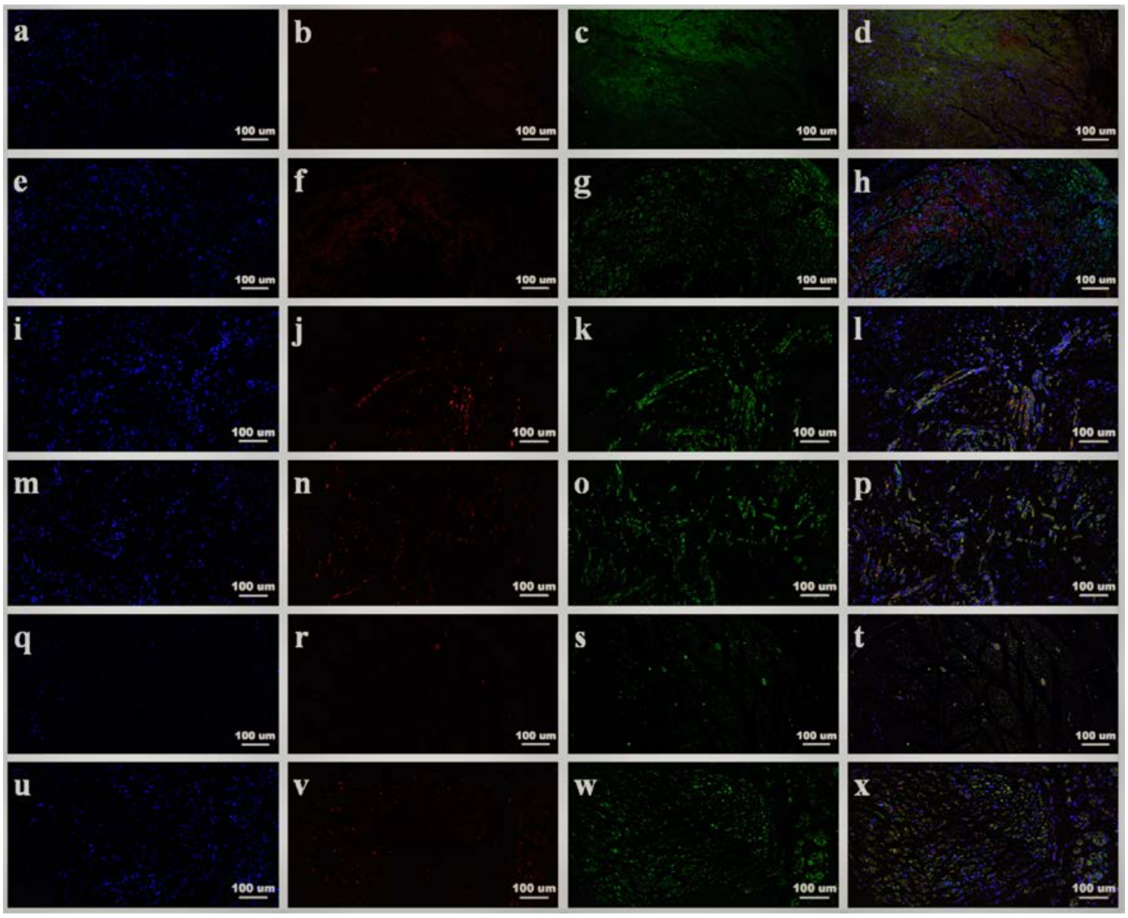

Supplementary Figure 12. Triple immunofluorescent staining of S100 and MBP of regenerated nerves at 6 weeks postoperatively. S100 (green), MBP (red) and nuclei (blue) were exhibited from different groups respectively. (**a-d**) SC loaded PDA/RGD-SG/PCL. (**e-h**) SC loaded PDA/RGD-MG/PCL. (**i-l**) PDA/RGD-SG/PCL. (**m-p**) PDA/RGD-MG/PCL. (**q-t**) PDA/RGD-PCL. (**u-x**) Autograft. The scale bar is 100 μm.

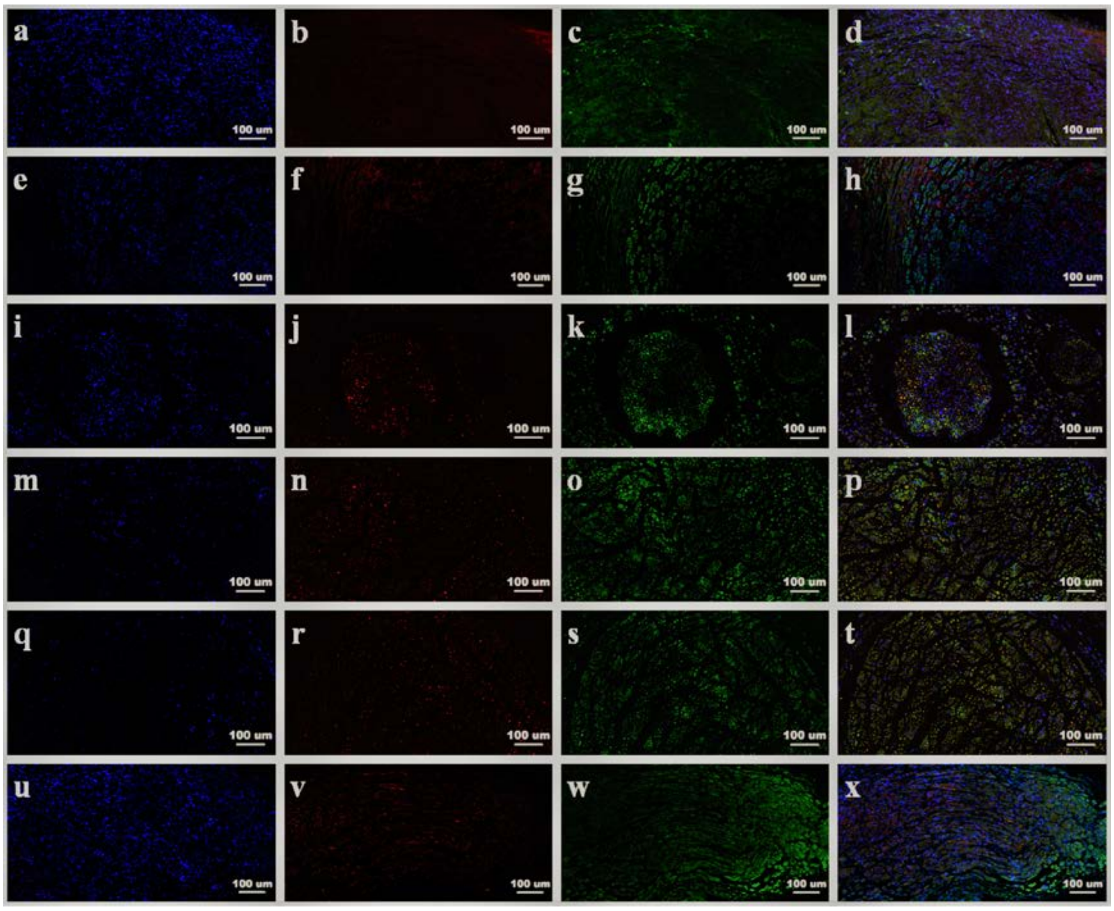

Supplementary Figure 13. Triple immunofluorescent staining of S100 and MBP of regenerated nerves at 12 weeks postoperatively. S100 (green), MBP (red) and nuclei (blue) were exhibited from different groups respectively. (**a-d**) SC loaded PDA/RGD-SG/PCL. (**e-h**) SC loaded PDA/RGD-MG/PCL. (**i-l**) PDA/RGD-SG/PCL. (**m-p**) PDA/RGD-MG/PCL. (**q-t**) PDA/RGD-PCL. (**u-x**) Autograft. The scale bar is 100 μm.

Certificate of Analysis

分析报告

**Product Name**	**Single layer graphene**
**Product number**	**HQNANO-GR-001**
**Batch number**	**02141115A**

**检测实验/TEST**	**要求和结果/SPECIFICATION**
Purity	>98wt%
Single rate	>97wt%
Thickness	0.5-3.0nm
Diameter	0.5-5μm
Layers	1-2

Supplementary Figure 14. Characteristics of single-layered graphene. The data were obtained from the product description provided by Suzhou Tanfeng Graphene Technology CO., Ltd (China). We displayed the purity, single rate, thickness, diameter, and layers of single-layered graphene.

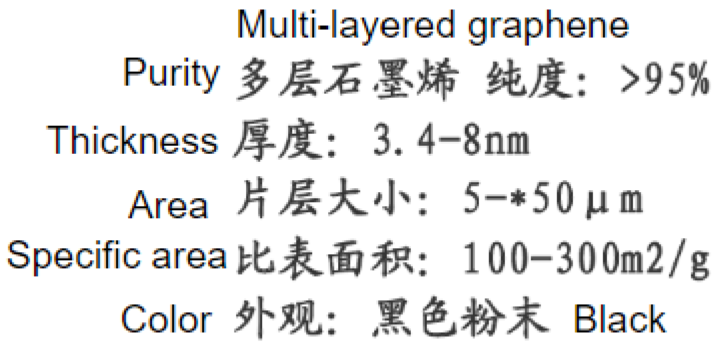

Supplementary Figure 15. Characteristics of multi-layered graphene. The data were obtained from the product description provided by Suzhou Tanfeng Graphene Technology CO., Ltd (China). We displayed the purity, thickness, area, specific area and color of multi-layered graphene.

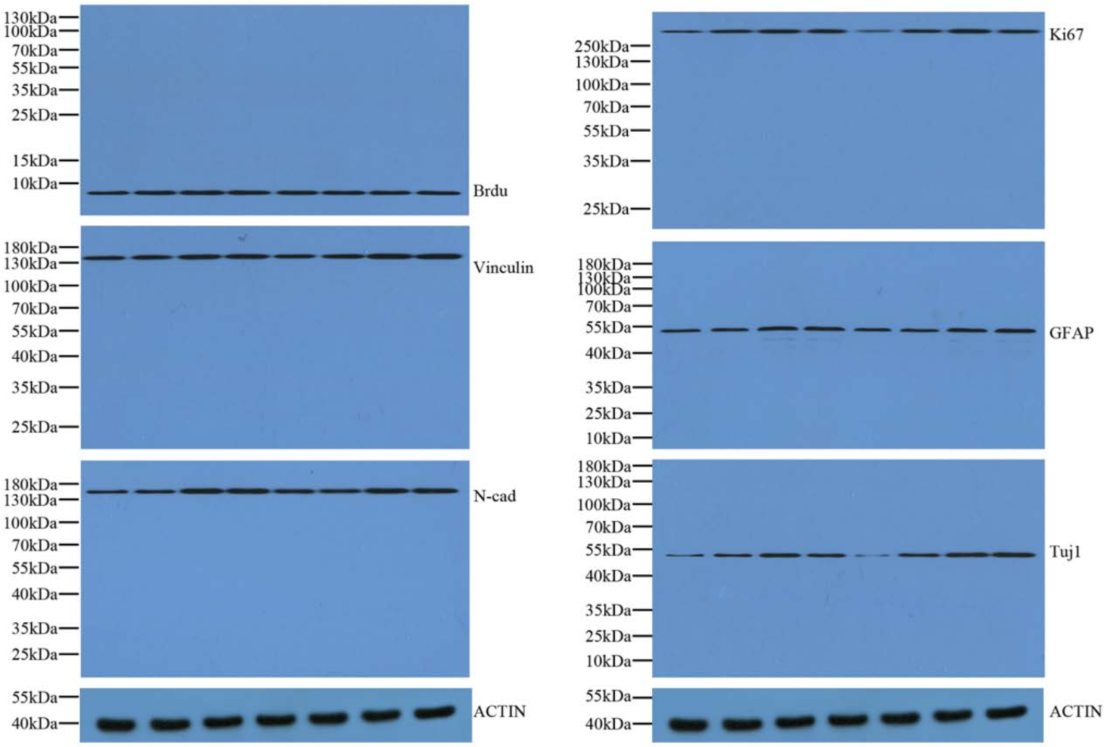

Supplementary Figure 16. Uncropped scans of Western blots (from Fig. 4).
